# Think about your friends and family: The disparate impacts of relationship-centered messages on privacy concerns, protective health behavior, and vaccination against Covid-19

**DOI:** 10.1371/journal.pone.0270279

**Published:** 2022-07-21

**Authors:** Clara K. Hanson, Kayuet Liu

**Affiliations:** 1 Department of Sociology, University of California, Los Angeles, Los Angeles, California, United States of America; 2 California Center for Population Research, University of California, Los Angeles, Los Angeles, California, United States of America; 3 Riken Center for Brain Science, Wako, Japan; Stevens Institute of Technology, UNITED STATES

## Abstract

**Objective:**

To understand which factors affect how willing people are to share their personal information to combat the Covid-19 pandemic, and compare them to factors that affect other public health behaviors.

**Method:**

We analyze data from three pre-registered online experiments conducted over eight months during the Covid-19 pandemic in the United States (April 3 2020 –November 25, 2020). Our primary analysis tests whether support for data sharing and intention to practice protective behavior increase in response to relationship-centered messages about prosociality, disease spread, and financial hardship. We then conduct a secondary correlational analysis to compare the demographic and attitudinal factors associated with willingness to share data, protective behavior, and intent to get vaccinated. Our sample (N = 650) is representative to socio-demographic characteristics of the U.S. population.

**Results:**

We find the altruistic condition increased respondents’ willingness to share data. In our correlational analysis, we find interactive effects of political ID and socio-demographic traits on likelihood to share data. In contrast, we found health behavior was most strongly associated with political ID, and intent to vaccinate was more associated with socio-demographic traits.

**Conclusions:**

Our findings suggest that some public health messaging, even when it is not about data sharing or privacy, may increase public willingness to share data. We also find the role of socio-demographic factors in moderating the effect of political party ID varies by public health behavior.

## Introduction

The Covid-19 pandemic has required sweeping social and behavioral changes. These changes are likely to continue as long as vaccines and treatments are not available globally, and new variants of the virus continue to emerge. Public health agencies have used a variety of messages to encourage beneficial behavior during this prolonged pandemic. Yet the pandemic has also made it clear that people, depending on their social contexts, vary in their judgment of whether to follow public health guidance.

Though public health surveillance has always relied on information collected from members of the public, the capacity of digital technology to support large-scale, real-time data collection meant willingness to share health information took on new importance. The promises of digital public health technologies are many, including personalized information about risk and exposure, and improvements to community outreach and overall care [1]. Both governments and private companies have developed new technology and disease surveillance programs with varying degrees of success [2]. However, a key challenge of these technologies is that their effectiveness depends on what proportion of the public participates, structure of disease spread, as well as material and social context [1, 3, 4]. These technologies also pose varying risks to privacy, which may not always be known to the public [2].

Given the varying benefits and risks of sharing health information, people vary in their decisions to share their information in the service of public health. Understanding how people make choices about their information, then, is an important component in the success of participatory surveillance programs. Initial surveys find there is relatively low public support of surveillance policies meant to decrease the spread of Covid-19, and suggest this is a barrier to their widespread adoption [5]. Relatively little is still known about how much people vary in their willingness to share information with public health officials, and why.

Likelihood to practice protective health behavior and get vaccinated are better-studied. Both are associated with a variety of demographic factors, attitudinal factors, social and cultural factors, as well as differences in access and knowledge [6, 7]. Yet studies of how to most effectively promote protective behavior and vaccination are ongoing. Studies of the role of altruism, for example, in the Covid-19 pandemic are mixed [8–10], as is previous research on altruism promoting messages about vaccinations and other diseases [11–13]. Though protective behavior and vaccination are the subject of a great deal of research, encouraging behavioral change and vaccination remain difficult problems.

One possible reason prior findings are mixed could be related to reference groups. Often, altruistic messages focus on how actions benefit society as a whole rather than highlighting benefits to people participants are connected to in real life. Studies have shown that social proximity to beneficiaries increases levels of altruism and reciprocity [14]. Moreover, social proximity alters perceptions of probabilities: people perceive events that happen in their social circle to be more likely to happen generally [15, 16]. Encouraging people to think about their immediate social networks may alter assessments of risk and increase the likelihood of respondents supporting public health measures. This theory is bolstered a growing recognition that tapping into one’s cognitive understanding of their personal network (“network cognition”) can produce different behavior [17]. However, this phenomenon has yet to be broadly applied to public health. Privacy behavior is highly context dependent, and so it may be especially responsive to subtle framing effects compared to the “actual” behavioral changes often studied in health communication (e.g. condom use, vaccinations) [18].

### Present study

The primary purpose of our study is to understand how data sharing behavior compares to more established behaviors like protective health behavior and vaccination. We investigate these differences using relationship-centered communication experiments and correlational analysis of our data. Relationship-centered messages ask respondents to consider the experiences of people they know as part of their messaging. This type of message applies network cognition theories that expect the perceptions people hold of their own social ties to affect their judgments of probabilities. In our experiments, we test whether these relationship-centered messages increase respondent support for public health. We tested the effect of three different relationship-centered messages: one message about protecting high-risk loved ones, one message about how a virus might spread through a respondent’s social network, and one message about the financial hardship loved ones may experience as a result of the pandemic.

We chose messages for our experimental conditions that appeared in communication about the pandemic during the periods we ran the experiment. This enabled us to explore the effect of salient messages as the social context of the pandemic changed. Altruistic and prosocial messages are common in prior research and have been applied to public health communication throughout this pandemic. Our message about protecting high-risk loved ones applies a version of this altruistic message. Prior research shows social network interaction affects risk perception [19]. Communication about Covid-19 often implicitly discusses interpersonal disease spread, but less often explicitly evokes the role of social network structure. The disease spread message explicitly connects disease spread with each respondent’s personal network. Finally, prior research finds perceptions of Covid-19 shaped economic anxiety [20]. Given the amount of attention paid to the economic impacts of the pandemic, we use the financial hardship condition to consider if economic anxiety also shapes Covid-19 behavior. We expected each message to increase respondents’ likelihood to practice protective behavior and their willingness to share data. We find a significant effect of the message about protecting high-risked loved ones on privacy behavior. We find no other significant effects of the experimental conditions on our outcome variables.

Our second purpose is to analyze the socio-demographic factors associated with willingness to share data. We compare these relationships to those correlated with likelihood to practice protective behavior and get vaccinated. We find privacy behavior is differently associated with demographic and attitudinal factors compared to protective behavior and vaccination. Consistent with [5], our study suggests willingness to share data is a distinct public health behavior. Our work underscores that people do not necessarily adopt all public health behaviors to the same extent.

## Methods

We test whether three relationship-centered messages increase public health outcomes: an altruistic message about protecting high-risk loved ones (“prosocial message”), a message about disease spread through one’s network (“disease spread message”), and a message about the economic effects of the pandemic (“hardship message”). In our pre-registered hypotheses (see S1 File, section 1), we expected each of the messages to increase support for information sharing and likelihood to engage in protective behavior compared to the controls.

We conducted a series of online survey experiments. For each experiment, respondents were randomly assigned to the experimental or control condition. Please see S1–S3 Figs for wording of each control and experimental condition. Within each experimental condition, respondents read a short text passage and answered a series of write-in questions. Within each control condition, respondents read a short informational passage about Covid-19 from the Centers for Disease Control. The passage includes information about Covid-19 symptoms, how to prevent the spread of the virus, and what to do if someone becomes sick. Respondents in the control condition then answered a series of write-in questions about contagious diseases.

Respondents were recruited through the research firm Qualtrics. All respondents were US residents over the age of 18, and the sample adhered to quotas for gender (50% male, 50% female), race (~66% non-Hispanic white, ~12% black, ~12% Hispanic, ~10% other), and education (50% some college or less, 50% associates and above). We use this quota sampling approach to collect a sample that corresponds to the sociodemographic characteristics of the US population. Attention and consistency checks ensured respondents read the material and gave consistent answers. Qualtrics panelists are subject to identity verification to prevent duplicate respondents. This research was approved by the Institutional Review Board (IRB) of the University of California, Los Angeles. The IRB granted a waiver of informed consent for this research.

### Experimental conditions

#### Prosocial message: Protecting high-risk loved ones

This condition encouraged respondents to think about protecting loved ones at high risk for serious illness from COVID-19. Respondents read an excerpt from the CDC’s website explaining COVID-19 with information about preventive health behavior. It also featured the phrase “Protect yourself, protect others” in bold. Respondents were then asked to think of two to five people at risk for serious illness from COVID-19, and list their relationship to them in a free response box. Respondents were then asked to think of two to five people who their friends or family would want to protect from COVID-19, and list them as well. Data for this condition and control were collected between April 3 to April 8, 2020. A total of 50 respondents were randomly assigned to the control, and 47 respondents were randomly assigned to the prosocial message. This condition considers whether an altruistic, network-based message increases preventive health behavior compared to a similar message without a network-based, altruistic prime.

#### Disease spread message: Imagining disease spread through one’s personal network

This condition asked respondents to imagine the spread of COVID-19 through their personal network. Respondents read an excerpt from a New York Times op-ed that described exposure to COVID-19 through a family member, and traced potential spread through the author’s personal network. Respondents were then asked to imagine disease spread through their own network by listing 2–5 pairs of people in written response boxes, with the first listed person being someone the respondent could contract COVID-19 from, and the second being someone the first listed person could contract COVID-19 from.

Respondents were not explicitly asked to imagine the disease spreading among their loved ones, though the examples given in the question suggested naming socially close people. Most respondents named some people they are socially close to (e.g. friend, mother), and some chose people they are socially further from (e.g. Uber driver, cashier). We coded responses according to their social closeness. Responses were considered “socially close” if they were one of the following: (1) a family relation, (2) a friend, or (3) were identified by name. Respondents who named at least one socially close person were considered suggestible to the prime. After responses were coded, 134 out of 148 responses met this criteria.

Data for this condition were collected in two waves: October 19–28, 2020, and November 20–25, 2020. A total of 102 respondents were randomly assigned to the control, and 148 respondents were randomly assigned to the disease spread message. This condition tests whether thinking about disease spread in terms of one’s network increases preventive health behavior compared to general information about disease.

#### Hardship message: Considering economic hardship caused by the pandemic

This condition encouraged respondents to think of people in their personal network who experienced financial hardship after the onset of the pandemic. In this condition, respondents read a news excerpt from the New York Times about unemployment claims in the United States. They were also asked to list their relationship to 2–5 people experiencing financial hardship whom they would like to help, and to briefly describe how that person had been affected by the pandemic in written response boxes. Data for this condition was collected from May 1 to May 11, 2020. A total of 73 respondents were randomly assigned to the control, and 69 respondents were randomly assigned to the hardship message. This condition tests whether thinking about secondary effects of addressing the pandemic in terms of one’s network increases preventive health behavior.

A limitation of these experiments is that we do not include control conditions of the same topic as the experimental conditions, but with general messages rather than relationship-centered messages. Instead, we compare our experiments to similar control conditions. We are thus unable to test whether using a relationship-centered message produces a stronger effect than discussing a topic generally. However, an advantage of this design is that conditions are more comparable to one another.

### Outcome measures

#### Data sharing

Respondents across surveys were asked how strongly they agreed or disagreed with their own data being collected for surveillance on a 5-point Likert scale. The questions addressed accessing phone location data, publicizing the identity and location of those diagnosed with COVID-19, and using tracking devices to enforce quarantine. There were five questions in total. These questions were drawn from policies discussed in or adopted by China, South Korea, Singapore, and the United States by the end of March 2020. Many of these measures would violate privacy norms in the US, where our data collection took place. However, surveying respondents on norm-violating policies allows us to measure the extent to which emergency circumstances change norms. Respondents’ answers to the five questions were averaged to create the data sharing variable. Low scores indicate a high support of privacy at the expense of surveillance, and high scores indicate high willingness to share information at the expense of privacy. Scores of this variable range from 1–5.

#### Protective behavior

This variable measures how likely respondents were to practice protective behavior on a 5-point Likert scale. In the disease spread and hardship message experiments, respondents were asked three questions: if they were likely to wear a face mask, step away if someone stood near them, and avoid crowded places in their everyday lives. In the prosocial message experiment, respondents were asked to imagine they exhibited symptoms of COVID-19, and then rated their likelihood to do three things: wear a face mask, self-isolate, and encourage others to “stop the spread.” The health behavior variable is the mean of each respondent’s answers. Scores of this variable range from 1–5.

#### Vaccination intent

Respondents surveyed between October and November 2020 were asked how strongly on a 5-point Likert scale they agreed with the statement: “I plan to get vaccinated for COVID-19 when a vaccine is approved by the FDA.” The response to this singular question measures intent to be vaccinated.

### Independent variables

#### Party identification (ID)

We used the partyid question of the General Social Survey. A binary variable was created by grouping respondents who identified as Democrats or as Democrat-leaning, and excluding those who identified as wholly independent or other.

#### Race

The race variables are self-identified and not mutually exclusive.

#### Education

Respondents are grouped by their highest level of education: a high school diploma, an associate’s degree or some college, a bachelor’s degree, or a graduate degree.

#### Racism and xenophobia

Respondents surveyed between October and November 2020 answered four questions of the explicit racial resentment scale [21] and seven questions to measure xenophobia [22]. Responses to each scale were highly positively correlated (*r*(411) = 0.659, *p* = 0.000). We averaged responses to make a single variable.

In our correlational analysis, we analyze the interaction between political ID and time, education, racism and xenophobia, gender, and age. Political ID was consistently predictive of behavior during this period of the pandemic [23, 24], and so it is a major focus of this analysis. We chose our other variables for the following reasons: the circumstances of the pandemic changed from month to month, and so we examined the association between our outcome variables and time. We included education, gender, and age because they are demographic factors that are often predictive of health behavior. Some people responded to the pandemic with racial animosity [25, 26], and so we also analyze the association with racism and xenophobia. Compositional differences and sample size preclude us from making comparisons about the interaction of race and party ID. The associations between race and our outcome variables are available in S3 Table.

## Results

**Fig 1:**
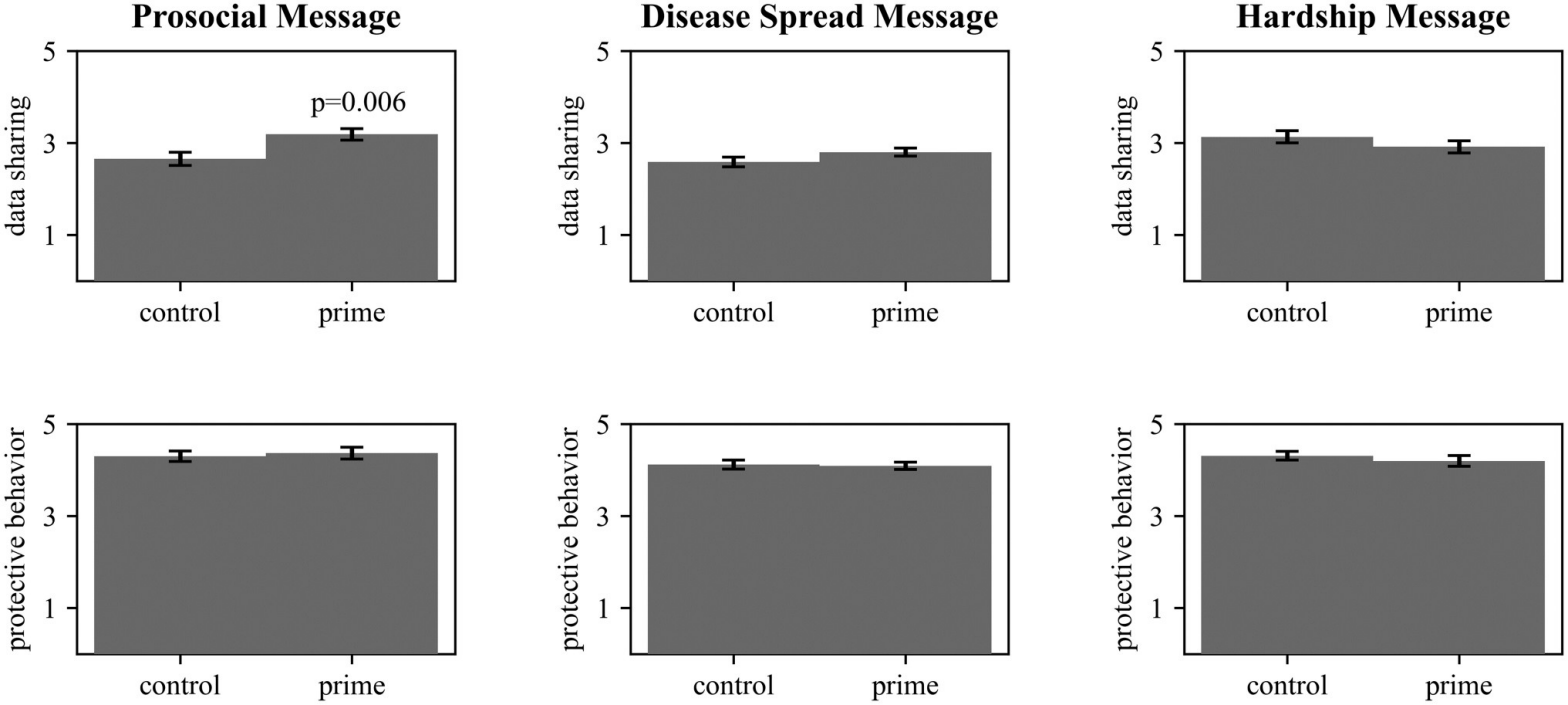
Effect of relationship-centered messages on data sharing and health behavior. Bars show mean response for data sharing (first row) and protective behavior (second row) among respondents assigned to the prosocial message (column 1), disease spread message (column 2), or hardship message (column 3). Error shows standard error of the mean. P-value measured by two-tailed t-test.

First, we analyze the effect of our experimental conditions on data sharing and protective behavior. We expected each of the personalized messages to increase respondents’ likelihood to share data and practice protective behavior. Respondents shown the prosocial message were significantly more likely to share data (*M* = 3.183, *SD* = 0.850) at the expense of privacy than respondents assigned to the control, *t*(95) = 2.809, *p* = 0.006, a difference of 0.535 points on average (see Fig 1). We did not find evidence the disease spread message significantly increased data sharing among all surveyed respondents (*M* = 2.797, *SD* = 1.033), *t*(248) = 1.595, *p* = 0.112. However, among respondents who imagined contracting Covid-19 from at least one socially-close contact, there was a marginal positive effect (*M* = 2.831, *SD* = 1.0144), *t*(234) = 1.829, *p* = 0.067. This provides weak evidence of an effect among those who were impressionable to the priming condition.

We do not find evidence the hardship message affected support for data sharing (*M* = 2.913, *SD* = 1.079), *t*(140) = -1.171, *p* = 0.243, nor evidence any of the messages affected likelihood to practice protective behavior: prosocial message: (*M* = 4.362, *SD* = 0.881), *t*(95) = -0.394, *p* = 0.694, disease spread message: (*M* = 4.086, *SD* = 0.945), *t*(248) = 0.232, *p* = 0.817, hardship message: (*M* = 4.193, *SD* = 0.952), *t*(68) = 0.774, *p* = 0.440. Respondents were asked about vaccination intent only in the disease spread message experiment, and there was no evidence of an effect of that message (*M* = 3.581, *SD* = 1.405), t(248) = 0.701, *p* = 0.484. See S2 Table for a full overview of all condition means and standard deviations.

There are multiple reasons why the messages in our experiment affected data sharing but not protective behavior. It is possible data sharing is more responsive to framing effects than protective behavior in general. It is also possible that different types of messages motivate different kinds of behavior. However, respondents were likely exposed to much more messaging about protective health behavior than data sharing prior to taking part in our study, and therefore may have had stronger priors for these behaviors. Data sharing may be more susceptible to subtle messaging in part because it is less-often the subject of directive messaging.

**Fig 2:**
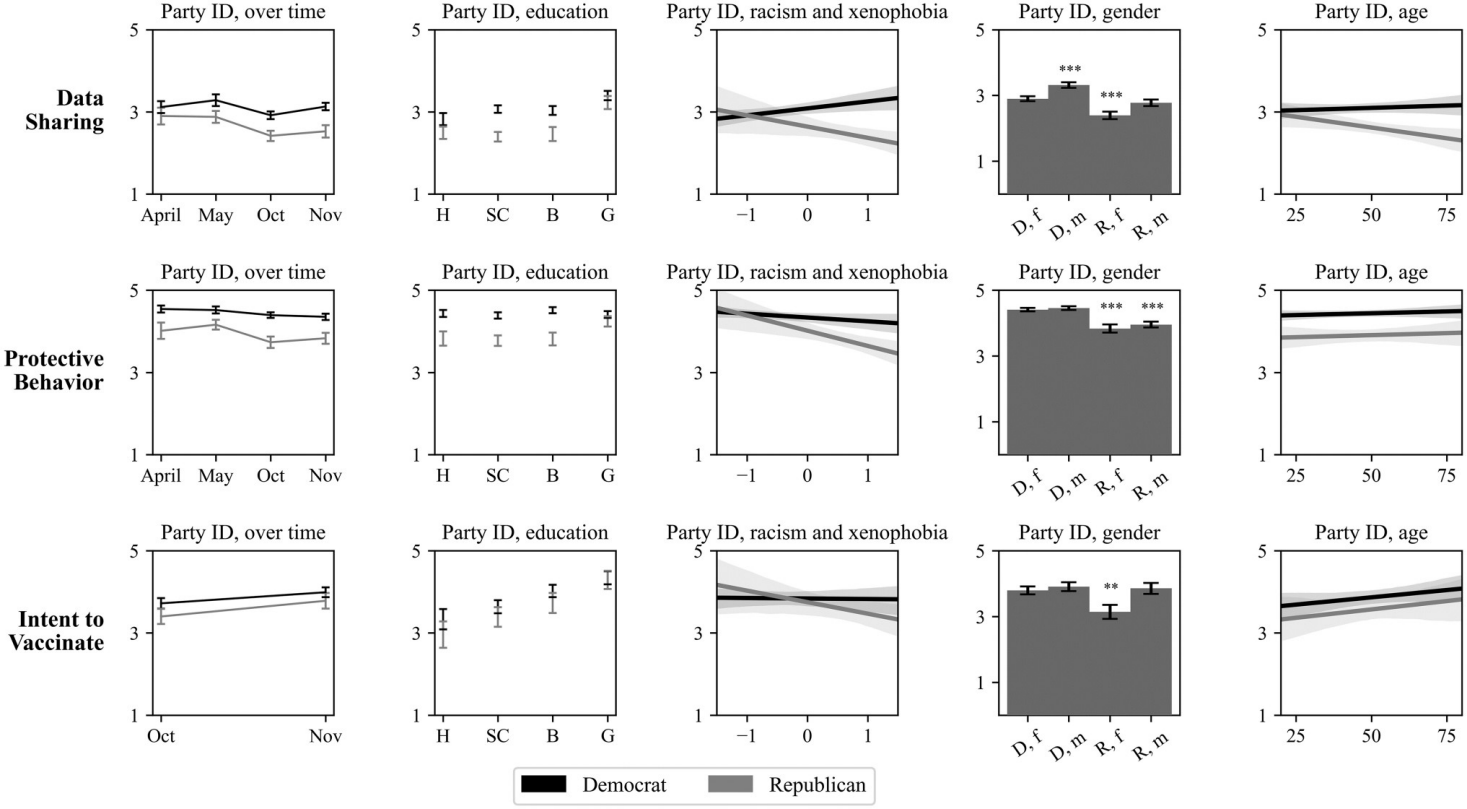
Association between independent variables and data sharing, health behavior, and vaccination by party ID. Figure shows relationship between month (column 1), education (column 2), racism and xenophobia (column 3), gender (column 4), and age (column 5) on the x axes and data sharing (first row), protective behavior (second row), and intent to vaccinate (third row) on the y axes. Estimates for Democrats (in black) and Republicans (in gray). Error shows standard error of the mean, and shaded area around the line of best fit represents the 95% confidence interval. The time scales in the first column correspond to the months we collected responses, and are not evenly distributed. The specific dates are: 4/3–4/8, 5/1–5/11, 10/19–10/28, 11/20–11/25. All data collected in 2020. Codes for the x-axis are as follows: H = high school diploma, SC = some college or associate’s degree, B = bachelor’s degree, G = graduate degree. D,f = Democrat and female, D,m = Democrat and male, R,f = Republican and female, R,m = Republican and male. *⟊ = p < 0*.*10*, * = p < 0.*05*, ** = p < 0.*01*, *** = p < 0.*001*. P-value measured by two-tailed t-test.

We perform a secondary analysis to consider the extent to which data sharing, protective behavior, and vaccination are predicted by similar traits. These variables are only weakly correlated with each other (S1 Table). They are associated with different demographic traits, particularly political ID (S3 Table). To better understand how political ID and other factors relate to each other, we explore interactions in the association between political ID and time, education, gender, age, and racism and xenophobia and our outcome variables in Fig 2. Since the respondents were randomly assigned to their experimental conditions and thus confounding by experimental conditions is unlikely, we pooled the subjects from all conditions in order to yield more stable estimates. Yet the results reported here should be still understood as general trends rather than as precise point estimates. See S4 Table for full set of regression results.

One’s likelihood to practice each behavior is significantly associated with political ID. While this could suggest simple polarization, our analysis suggests the relationship between political ID and public health behavior is sometimes moderated by other factors. The factors that moderate political ID vary across our three outcome variables. This means the relationship between party ID and behavior varies both by respondent and by type of health behavior.

Specifically, both political ID and socio-demographic traits are related to data sharing: Democratic respondents reported higher likelihood to share data than Republican respondents overall, but the likelihood also varies by gender, with men from either party more likely to share data than women. The likelihood to share data declined with age among Republicans but not Democrats. Likelihood to share data is also polarized among respondents with some college or a bachelor’s degree, but not among respondents with a high school diploma or a graduate degree (top panel).

In comparison, Democrats reported a significantly higher likelihood to practice preventive health behavior than Republicans regardless of gender and age (middle panel). With the exception that Republicans with a graduate degree have similar levels of health behavior compared to their Democrat counterparts, there is no evidence education moderates the association between party ID and health behavior.

The interaction of racism and xenophobia with political ID was consistent for data sharing and protective behavior. Fig 2 shows that both data sharing and health behavior were more strongly associated with racism and xenophobia among Republican respondents than among Democrat respondents.

There is no evidence political ID moderates the relationship between either age or education with our dependent variables. Older respondents were marginally more likely to intend to get vaccinated than younger respondents, and respondents with higher levels of education are significantly more likely to get vaccinated than those with lower levels of education. In our sample, women were significantly less likely to get vaccinated than men, but this difference is explained by political ID; there is no significant difference in intent to vaccinate among Democrat women, Democrat men, and Republican men, but Republican women reported a significantly lower likelihood to intend to be vaccinated.

## Discussion

Despite the possibilities of digital technology for public health surveillance, initial measures of public support for these technologies was low [5]. Little is known about the mechanisms that cause people to support data sharing for public health. The purpose of our analysis is to contribute to understandings of the factors that affect how willing people are to share their personal information to support public health. We first analyze the effect of three relationship-centered messages to explore how personalized framing of the pandemic affects willingness to share data. We then conduct a correlational analysis to understand the relationship between political ID, socio-demographic characteristics, and three different kinds of public health behavior.

In our experiments, we found the message about health risks to respondent’s loved ones increased respondent support for data sharing, but the messages about economic hardship did not. We found weak evidence that messages about risk of disease spread in one’s community may increase willingness to share data among some people. Compared to data sharing, other protective health behaviors like wearing a mask were not affected by such relationship-centered messages. It is difficult to change opinions about a salient topic, and so this may explain why our priming materials changed responses to privacy questions but not those for other health behaviors. These results suggest that how public health messages are framed can affect how willing people are to share their information. However, our experiments are inconclusive about the distinct effects of context and message content, and the effects we find are small. While our work asks novel questions, we suggest replication and expansion of our work is needed to understand what makes people willing to share their personal information and measure the effectiveness of messaging campaigns for behavioral change.

The protective health behaviors we studied have different patterns of association with socio-demographic variables and attitudes. Specifically, consistent with prior research about the role of partisanship in the Covid-19 pandemic, we find protective behavior is primarily associated with political party ID [24]. Intent to be vaccinated was correlated with age, level of education, and an interaction between gender and political party ID. This is also consistent with earlier trends in vaccination during the Covid-19 pandemic [27] and prior to it [28]. Finally, we found the association between political party ID and data sharing was dependent on several different socio-demographic characteristics, including: age, education, and gender.

Attitudes toward privacy in public health contexts are understudied, and many of the findings are inconsistent [29]. This study is consistent with the finding that Democrats tend to be more supportive of secondary use of data than Republicans [30]. It is also consistent with a study of privacy in the Covid-19 pandemic that found men, people with higher levels of education, and Democrats were more supportive of a variety of types of surveillance [5]. This analysis adds that the interaction between socio-demographic characteristics and political ID is important in the prediction of data sharing. This observation is not obvious given that protective health behavior is more straightforwardly polarized.

Our study contributes two additional findings. First, our correlational analysis finds racism and xenophobia are correlated with protective health behavior and data sharing after controlling for political ID. This suggests resentment against outgroups could decrease practicing important public health behavior. This provides additional support for prior findings about the role of xenophobia, racism, and nationalism in responses to the Covid-19 pandemic [25, 26].

Second, our experiments explored whether relationship-centered messages can affect respondent behavior. There is a growing recognition that tapping into a person’s cognitive understanding of their personal network can produce different behavior [17]. This phenomenon has yet to be broadly applied to public health, even though it may be especially relevant because it can shape perceived risks and benefits of interventions [31, 32]. In this analysis, we are unable to analyze whether relationship-centered messages provoke stronger responses than more general messages on the same topic. However, we hope this preliminary analysis inspires additional research on the role of network-cognition in public health communication.

### Limitations

This study has the following limitations. First, it uses only one message type and one control condition per experiment. The messages and the control vary in content, and so we cannot rule out the possibility that the experimental results are the consequence of particular features of these conditions as opposed to the result of the general messages we tested. Second, each wave of the survey tested the effect of only one experimental condition and one priming condition. The data was collected in four waves over eight months during a time of rapid societal change, and so this design limits the comparability of our experimental results and introduces time as a confounding variable—although the timeliness of our data collection provides information from critical points during the pandemic. Replicating this study by running all three experiments at the same time with additional conditions would clarify the different effects of context, message content, and level of personalization.

An additional limitation is the potential of self-selection into experimental conditions. Our experimental design limits the potential for self-selection by randomly assigning participants to conditions. It is possible, albeit unlikely, that respondents differentially opted-out of conditions. This would limit our ability to make causal claims from our experiment. However, our findings on socio-demographic correlates of behavior are compatible with surveys of larger samples, indicating consistency between our findings and broader behavioral trends.

### Implications

This work provides some initial considerations for public health communications strategies to promote data sharing. Traditional altruistic public health messages to encourage data sharing may become more effective if they can incorporate relationship-centered messages. In addition, we found demographic variables were differently associated with data sharing, protective health behavior, and vaccination. Our findings suggest groups that require the most intervention to adopt some public health behaviors (e.g. vaccination) may also be different from those who are most willing to share data with public health authorities. The findings of this study support that such selectivity into databases that rely on voluntary data sharing must be taken into account when authorities try to generalize findings from such databases to members of groups most resistant to behavioral change.

## Supporting information

S1 File (DOCX)

S1 Table Correlations among dependent variables. (DOCX)

S2 Table Mean and standard deviation of outcome variables across conditions. (DOCX)

S3 Table Distribution of demographic and attitudinal variables across dependent variables. (DOCX)

S4 Table Interactions between key demographic traits and Party ID on dependent variables. (DOCX)

S1 Fig Conditions for prosocial message experiment. (DOCX)

S2 Fig Conditions for disease spread message experiment. (DOCX)

S3 Fig Conditions for hardship message experiment. (DOCX)
