# Supplementary material for: Think about your friends and family: The disparate impacts of relationship-centered messages on privacy concerns, protective health behavior, and vaccination against Covid-19
Source: PLoS One. 2022 Jul 21;17(7):e0270279. doi: 10.1371/journal.pone.0270279 (PMC9302763; doi:10.1371/journal.pone.0270279)
Supplement: S1 File — (DOCX) [file pone.0270279.s001.docx]

**Supplemental Materials**

**Section 1: Pre-registration Materials**

<https://aspredicted.org/blind.php?x=/PKN_JZT> (registered on (04/03/2020)

<https://aspredicted.org/blind.php?x=/TMH_X29> (registered on 05/05/2020)

<https://aspredicted.org/blind.php?x=/RTZ_ZP4> (registered on 10/26/2020)

Above are links to our pre-registration documents for peer-review on AsPredicted.org. All pre-registrations took place prior to data collection or access to the data.
