# Supplementary material for: Think about your friends and family: The disparate impacts of relationship-centered messages on privacy concerns, protective health behavior, and vaccination against Covid-19
Source: PLoS One. 2022 Jul 21;17(7):e0270279. doi: 10.1371/journal.pone.0270279 (PMC9302763; doi:10.1371/journal.pone.0270279)
Supplement: S1 Table — (DOCX) [file pone.0270279.s002.docx]

**Section 2: Supplemental tables**

Table A1: Correlations among dependent variables

|  | **Data Sharing** | **Protective Behavior** | **Intent to be Vaccinated** |
| --- | --- | --- | --- |
| **Data Sharing** |  |  |  |
| **Health Behavior** | *r*(650) = 0.327,  *p* = 0.000 |  |  |
| **Intent to be Vaccinated** | *r*(411) = 0.327,  *p* = 0.000 | *r*(411) = 0.302,  *p* = 0.000 |  |
