# Supplementary material for: Think about your friends and family: The disparate impacts of relationship-centered messages on privacy concerns, protective health behavior, and vaccination against Covid-19
Source: PLoS One. 2022 Jul 21;17(7):e0270279. doi: 10.1371/journal.pone.0270279 (PMC9302763; doi:10.1371/journal.pone.0270279)
Supplement: S2 Table — (DOCX) [file pone.0270279.s003.docx]

Table A2: Mean and Standard Deviation of Outcome Variables Across Conditions

|  | **Data Sharing** | | **Protective Behavior** | | **Intent to Vaccinate** | |
| --- | --- | --- | --- | --- | --- | --- |
|  | **Mean** | **SD** | **Mean** | **SD** | **Mean** | **SD** |
| **Prosocial Control** | 2.648 | 1.012 | 4.295 | 0.785 | - | - |
| **Prosocial Prime** | 3.183 | 0.850 | 4.362 | 0.881 | - | - |
|  |  |  |  |  |  |  |
| **Hardship Control** | 3.129 | 1.114 | 4.306 | 0.779 | - | - |
| **Hardship Prime** | 2.913 | 1.079 | 4.193 | 0.952 | - | - |
|  |  |  |  |  |  |  |
| **Network Control 1** | 2.496 | 1.013 | 3.845 | 1.162 | 3.393 | 1.358 |
| **Network Prime 1** | 2.797 | 1.036 | 4.118 | 0.923 | 3.566 | 1.389 |
|  |  |  |  |  |  |  |
| **Network Control 2** | 2.691 | 1.083 | 4.442 | 0.617 | 4.087 | 1.262 |
| **Network Prime 2** | 2.797 | 1.036 | 4.051 | 0.972 | 3.597 | 1.431 |
