# Supplementary material for: Think about your friends and family: The disparate impacts of relationship-centered messages on privacy concerns, protective health behavior, and vaccination against Covid-19
Source: PLoS One. 2022 Jul 21;17(7):e0270279. doi: 10.1371/journal.pone.0270279 (PMC9302763; doi:10.1371/journal.pone.0270279)
Supplement: S3 Table — (DOCX) [file pone.0270279.s004.docx]

Table A3: Distribution of demographic and attitudinal variables across dependent variables

|  | **Data Sharing** | | | **Protective Behavior** | | | **Intent to Vaccinate** | | |
| --- | --- | --- | --- | --- | --- | --- | --- | --- | --- |
| Variable | **n** | **% (95% CI)** | ***P*** | **n** | **% (95% CI)** | ***P*** | **n** | **% (95% CI)** | ***P*** |
|  |  |  |  |  |  |  |  |  |  |
| Age | 650 | -0.0026, (-0.007, 0.002) | 0.262 | 650 | -0.0007, (-0.005, 0.003) | 0.737 | 411 | 0.0076, (0.000, 0.016) | 0.06 |
|  |  |  |  |  |  |  |  |  |  |
| Gender |  |  |  |  |  |  |  |  |  |
| Male (ref) | 327 | 2.984, (1.899, 4.070) |  | 327 | 4.150, (3.214, 5.086) |  | 206 | 3.806, (2.437, 5.175) |  |
| Female | 323 | 2.663, (1.700, 3.626) | 0.000 | 323 | 4.218, (3.336, 5.100) | 0.347 | 205 | 3.434, (2.030, 4.838) | 0.007 |
|  |  |  |  |  |  |  |  |  |  |
| Race |  |  |  |  |  |  |  |  |  |
| White (ref) | 488 | 2.784, (1.718, 3.850) |  | 488 | 4.152, (3.212, 5.092) |  | 316 | 3.626, (2.187, 5.065) |  |
| Black | 88 | 2.950, (1.984, 3.916) | 0.173 | 88 | 4.306, (3.488, 5.125) | 0.151 | 49 | 3.204, (1.761, 4.647) | 0.057 |
| Asian | 62 | 3.116, (2.307, 3.925) | 0.018 | 62 | 4.308, (3.542, 5.074) | 0.212 | 36 | 3.917, (2.921, 4.913) | 0.240 |
| Other | 29 | 2.448, (1.526, 3.370) | 0.098 | 29 | 3.796, (2.646, 4.944) | 0.051 | 24 | 3.500, (2.149, 4.851) | 0.677 |
|  |  |  |  |  |  |  |  |  |  |
| Education |  |  |  |  |  |  |  |  |  |
| High School (ref) | 129 | 2.634, (1.630, 3.638) |  | 129 | 4.109, (3.176, 5.042) |  | 79 | 3.063, (1.575, 4.551) |  |
| Some College | 233 | 2.713, (1.704, 3.722) | 0.474 | 233 | 4.098, (3.121, 5.075) | 0.916 | 150 | 3.453, (2.013, 4.893) | 0.055 |
| Bachelor's Degree | 154 | 2.777, (1.743, 3.811) | 0.243 | 154 | 4.261, (3.393, 5.129) | 0.157 | 104 | 3.808, (2.546, 5.07) | 0.000 |
| Graduate School | 127 | 3.268, (2.258, 4.276) | 0.000 | 127 | 4.303, (3.477, 5.102) | 0.074 | 74 | 4.243, (3.135, 5.351) | 0.000 |
|  |  |  |  |  |  |  |  |  |  |
| Party ID |  |  |  |  |  |  |  |  |  |
| Democrat (ref) | 307 | 3.085, (2.104, 4.066) |  | 307 | 4.429, (3.736, 5.122) |  | 197 | 3.843, (2.589, 5.097) |  |
| Republican | 201 | 2.624, (1.549, 3.699) | 0.000 | 201 | 3.905, (2.880, 4.930) | 0.000 | 125 | 3.568, (2.069, 5.067) | 0.077 |
| Other/Independent | 142 | 2.546, (1.570, 3.522) | 0.000 | 142 | 4.048, (3.041, 5.055) | 0.000 | 89 | 3.202, (1.739, 4.665) | 0.000 |
|  |  |  |  |  |  |  |  |  |  |
| Racism and Xenophobia | 411 | -0.1480, (-0.246, -0.050) | 0.003 | 411 | -0.2769, (-0.364, -0.190) | 0.000 | 411 | -0.1535, (-0.288, -0.019) | 0.026 |
|  |  |  |  |  |  |  |  |  |  |
