# Supplementary material for: Think about your friends and family: The disparate impacts of relationship-centered messages on privacy concerns, protective health behavior, and vaccination against Covid-19
Source: PLoS One. 2022 Jul 21;17(7):e0270279. doi: 10.1371/journal.pone.0270279 (PMC9302763; doi:10.1371/journal.pone.0270279)
Supplement: S4 Table — (DOCX) [file pone.0270279.s005.docx]

Table A4: Interactions between key demographic traits and Party ID on dependent variables

|  |  | **Data Sharing** | | **Protective Behavior** | | **Intent to Vaccinate** | |
| --- | --- | --- | --- | --- | --- | --- | --- |
|  | **Variable** | **% (95% CI)** | ***P*** | **% (95% CI)** | ***P*** | **% (95% CI)** | ***P*** |
|  |  |  |  |  |  |  |  |
| **Model 1** | Time | -0.0348, (-0.145, 0.075) | 0.535 | -0.0704, (-0.161, 0.020) | 0.127 | 0.2693, (-0.110, 0.649) | 0.164 |
|  | Republican | -0.0950, (-0.634, 0.444) | 0.729 | -0.3845, (-0.829, 0.060) | 0.090 | -0.6573, (-2.784, 1.469) | 0.543 |
|  | Republican * Time | -0.1312, (-0.314, 0.051) | 0.158 | -0.0498, (-0.200, 0.101) | 0.515 | 0.1126, (-0.498, 0.723) | 0.717 |
|  | Intercept | 3.1813, (2.855, 3.508) | 0.000 | 4.6246, (4.356, 4.893) | 0.000 | 2.9118, (1.586, 4.238) | 0.000 |
|  |  |  |  |  |  |  |  |
| **Model 2** | Education | 0.1610, (0.050, 0.272) | 0.005 | 0.0098, (-0.083, 0.103) | 0.836 | 0.3436, (0.160, 0.527) | 0.000 |
|  | Republican | -0.6673, (-1.134, -0.200) | 0.005 | -0.8515, (-1.243, -0.460) | 0.000 | -0.4591, (-1.242, 0.324) | 0.249 |
|  | Education * Republican | 0.0833, (-0.089, 0.256) | 0.342 | 0.1293, (-0.015, 0.274) | 0.079 | 0.0865, (-0.206, 0.379) | 0.561 |
|  | Intercept | 2.6818, (2.378, 2.986) | 0.000 | 4.4067, (4.152, 4.661) | 0.000 | 2.9708, (2.474, 3.467) | 0.000 |
|  |  |  |  |  |  |  |  |
| **Model 3** | Racism & Xenophobia | 0.1688, (0.021, 0.317) | 0.026 | -0.0928, (-0.224, 0.038) | 0.165 | 3.8376, (3.629, 4.046) | 0.908 |
|  | Republican | -0.4429, (-0.709, -0.177) | 0.001 | -0.3202, (-0.556, -0.085) | 0.008 | -0.0122, (-0.218, 0.194) | 0.636 |
|  | Republican*Racism & Xenophobia | -0.4425, (-0.703, -0.182) | 0.001 | -0.2783, (-0.509, -0.047) | 0.018 | -0.0892, (-0.459, 0.281) | 0.147 |
|  | Intercept | 3.0848, (2.935, 3.234) | 0.000 | 4.3351, (4.203, 4.468) | 0.000 | -0.2679, (-0.631, 0.095) | 0.000 |
|  |  |  |  |  |  |  |  |
|  | Female | -0.4139, (-0.640, -0.188) | 0.000 | -0.0482, (-0.238, 0.141) | 0.617 | -0.1142, (-0.493, 0.265) | 0.553 |
|  | Republican | -0.5401, (-0.785, -0.295) | 0.000 | -0.5041, (-0.710, -0.298) | 0.000 | -0.0536, (-0.470, 0.363) | 0.800 |
|  | Female*Republican | 0.0325, (-0.331, 0.396) | 0.860 | -0.0699, (-0.375, 0.235) | 0.652 | -0.5991, (-1.212, 0.013) | 0.055 |
|  | Intercept | 3.3139, (3.146, 3.482) | 0.000 | 4.4556, (4.314, 4.597) | 0.000 | 3.9070, (3.623, 4.191) | 0.000 |
|  |  |  |  |  |  |  |  |
