# Supplementary material for: Think about your friends and family: The disparate impacts of relationship-centered messages on privacy concerns, protective health behavior, and vaccination against Covid-19
Source: PLoS One. 2022 Jul 21;17(7):e0270279. doi: 10.1371/journal.pone.0270279 (PMC9302763; doi:10.1371/journal.pone.0270279)
Supplement: S1 Fig — (DOCX) [file pone.0270279.s006.docx]

Section 3: Experimental Conditions

Fig A1: Conditions for Prosocial Message Experiment

CONDITION 1

Start of Block: Condition 1

| 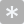 |
| --- |

Q4 Can you think of other contagious diseases that, like coronavirus, are contagious and spread from person to person? 


**Name up to 5 other contagious diseases that spread from person to person.**

________________________________________________________________

| 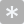 |
| --- |

Q55 Can you think of symptoms of coronavirus?


**Name up to 5 symptoms of coronavirus you can remember.**

________________________________________________________________

| Page Break |  |
| --- | --- |

Q5 **Please review the following information about coronavirus, excerpted from the CDC.**
 COVID-19 is a new disease, caused by a novel (or new) coronavirus that has not previously been seen in humans. 

 Current symptoms reported for patients with COVID-19 have included mild to severe respiratory illness with fever, cough, and difficulty breathing. 

 The best way to prevent illness is to avoid being exposed to this virus. The virus is thought to spread mainly from person-to-person.

 **Protect yourself, protect others**
 Stay home if you are sick, except to get medical care.

 Cover your mouth and nose with a tissue when you cough or sneeze or use the inside of your elbow.

 Wash your hands often with soap and water for at least 20 seconds especially after you have been in a public place, or after blowing your nose, coughing, or sneezing.

 Put distance between yourself and other people if COVID-19 is spreading in your community.

 If you are sick: You should wear a face mask when you are around other people and before you enter a healthcare provider's office.

| 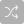 |
| --- |

Q6 According to the information above, which of the following IS NOT true?

- COVID-19 has not previously been seen in humans (1)
- The best way to prevent illness is to avoid being exposed to COVID-19 (2)
- You should wear a face mask around others if you are sick (3)
- If you use hand sanitizer, it should be 95% alcohol (4)

End of Block: Condition 1

CONDITION 2

Start of Block: Condition 2

| 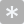 |
| --- |

Q7 Some people are at high risk for serious illness from coronavirus, including **older adults** and people with **chronic conditions** like **diabetes** and **heart disease**.


Can you think of anyone you'd want to protect from coronavirus?


**Please list your relationship to up to 5 people you'd want to protect** (ex. "dad," "friend," or "wife")

________________________________________________________________

| 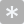 |
| --- |

Q8 Can you think of anyone your friends or family would want to protect from coronavirus? 


**Please list your relationship to up to 5 people your friends or family would want to protect** (ex. "friend's grandfather," "mom's coworker")

________________________________________________________________

| Page Break |  |
| --- | --- |

Q9 **Please review the following information about coronavirus, excerpted from the CDC.**

 **Remember that people you care about may be at higher risk for serious illness from coronavirus, including older adults and people with chronic conditions like diabetes and heart disease.**

 COVID-19 is a new disease, caused by a novel (or new) coronavirus that has not previously been seen in humans. 

 Current symptoms reported for patients with COVID-19 have included mild to severe respiratory illness with fever, cough, and difficulty breathing. 

 The best way to prevent illness is to avoid being exposed to this virus. The virus is thought to spread mainly from person-to-person.

 **Protect yourself, protect others**

 Stay home if you are sick, except to get medical care.

 Cover your mouth and nose with a tissue when you cough or sneeze or use the inside of your elbow.

 Wash your hands often with soap and water for at least 20 seconds especially after you have been in a public place, or after blowing your nose, coughing, or sneezing.

 Put distance between yourself and other people if COVID-19 is spreading in your community.

 If you are sick: You should wear a face mask when you are around other people and before you enter a healthcare provider's office.

| 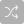 |
| --- |

Q10 According to the information above, which of the following IS NOT true?

- COVID-19 has not previously been seen in humans (1)
- The best way to prevent illness is to avoid being exposed to COVID-19 (2)
- You should wear a face mask around others if you are sick (3)
- If you use hand sanitizer, it should be 95% alcohol (4)
