# Supplementary material for: Think about your friends and family: The disparate impacts of relationship-centered messages on privacy concerns, protective health behavior, and vaccination against Covid-19
Source: PLoS One. 2022 Jul 21;17(7):e0270279. doi: 10.1371/journal.pone.0270279 (PMC9302763; doi:10.1371/journal.pone.0270279)
Supplement: S2 Fig — (DOCX) [file pone.0270279.s007.docx]

Fig A2: Conditions for Disease Spread Message Experiment

**Close Network Prime (disease spread prime)**

Start of Block: Close-Network Prime I

Q1 **You’ve been randomly assigned to read an excerpt of an article from the New York Times about Covid-19. Please read it and answer the following questions.**

My husband mentioned that his cousin S., who lives far away, would be visiting the area and wanted to come over for dinner in our backyard, and I paused.

“Don’t forget, my parents come next week,” I said.

But by the time the day came for S.’s visit, the desire for normalcy had pushed past thoughts of safety, and she arrived wearing a mask. She spent the next couple of hours in our backyard eating takeout Thai and talking without a mask. Then she put it back on as she left.

We got the text on the following Tuesday that S., who asked that only her first initial be used, had tested positive for the coronavirus. 

It was left to us — the 17 people whom S. had come into direct contact with between the date of the test and the result, and the many more who had come in contact with us and then with others — to do it ourselves. That came to almost 70 people.

It’s worth exploring what is happening across the country, and why it is likely to get worse, as schools and churches and bars and restaurants snap open and close while the virus continues to fester. My own family is white and privileged; we have access to doctors and sick days and smartphones. I can’t imagine how much worse our situation would be without those things.

prime1_attention According to the article, how many people did S. come into direct contact with between getting a test and getting the test result?

- 2 (1)
- 17 (2)
- 25 (3)
- 70 (4)

| 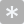 |
| --- |

prime1_boxes We want to understand your perception of risk in your daily life. 
**If you were to contract Covid-19, who do you think you’re most likely to get it from?**
In the boxes below, please list your relationship to 2-5 people who you could catch Covid-19 from (ex. “sister” or “friend”).
Then think about who they could catch Covid-19 from (ex. “friend” or “her husband”).

- 1a. Who could you catch Covid from? (1) ________________________________________________
- 1b. Who might they catch it from? (2) ________________________________________________
- 2a. Who could you catch Covid from? (3) ________________________________________________
- 2b. Who might they catch it from? (4) ________________________________________________
- 3a. Who could you catch Covid from? (5) ________________________________________________
- 3b. Who might they catch it from? (6) ________________________________________________
- 4a. Who could you catch Covid from? (7) ________________________________________________
- 4b. Who might they catch it from? (8) ________________________________________________
- 5a. Who could you catch Covid from? (9) ________________________________________________
- 5b. Who might they catch it from? (10) ________________________________________________

End of Block: Close-Network Prime I

Far Network Prime (not analyzed in this study)

*Note: This is a secondary control condition that was only included in the disease spread experiment. In this paper, we analyze only the control condition that was common across our experiments. We do not analyze this condition in the paper because it is beyond the scope of our analysis.*

Start of Block: Far-Network Prime I

Q5 You’ve been randomly assigned to read an excerpt of an article from the New York Times about Covid-19. Please read it and answer the following questions.

Singapore has seen a surge of coronavirus cases among migrant workers, after months of successfully controlling the outbreak. As of Tuesday, coronavirus cases linked to migrant worker dormitories accounted for 88 percent of Singapore’s 14,446 cases, including more than 1,400 new cases in a single day.

Many migrant workers live in packed dormitories on the outskirts of the city. These dormitories can house up to 20 people per room, making it almost impossible to follow social distancing guidelines. Migrant workers around the world have been among the most vulnerable groups affected by the pandemic.

S11, a dormitory with the largest cluster of the coronavirus in Singapore, houses more than 2,200 people who are infected. The dormitory has a capacity of over 10,000.

prime2_attention According to the article, what percentage of Covid-19 cases in Singapore are linked to migrant worker dormitories?

- 10% (1)
- 27% (2)
- 55% (3)
- 88% (4)

| Page Break |  |
| --- | --- |

prime2_boxes We want to understand your perception of risk in your daily life. 
**If you were to contract Covid-19, who do you think you’re most likely to get it from?**
In the boxes below, please list your relationship to 2-5 people who you could catch Covid-19 from (ex. “grocery cashier,” or “student”).
Then think about who they could catch Covid-19 from (ex.  “coworker” or “bus passengers”).

- 1a. Who could you catch Covid from? (1) ________________________________________________
- 1b. Who might they catch it from? (2) ________________________________________________
- 2a. Who could you catch Covid from? (3) ________________________________________________
- 2b. Who might they catch it from? (4) ________________________________________________
- 3a. Who could you catch Covid from? (5) ________________________________________________
- 3b. Who might they catch it from? (6) ________________________________________________
- 4a. Who could you catch Covid from? (7) ________________________________________________
- 4b. Who might they catch it from? (8) ________________________________________________
- 5a. Who could you catch Covid from? (9) ________________________________________________
- 5b. Who might they catch it from? (10) ________________________________________________

End of Block: Far-Network Prime I

Control Condition

Start of Block: Control Condition I

Q8 Please review the following information about Covid-19, excerpted from the CDC.

 COVID-19 is a new disease, caused by a novel (or new) coronavirus that has not previously been seen in humans.     

 Current symptoms reported for patients with COVID-19 have included mild to severe respiratory illness with fever, cough, and difficulty breathing.     

 The best way to prevent illness is to avoid being exposed to this virus. The virus is thought to spread mainly from person-to-person.    

 Protect yourself, protect others    

 Stay home if you are sick, except to get medical care.    

 Cover your mouth and nose with a tissue when you cough or sneeze or use the inside of your elbow.    

 Wash your hands often with soap and water for at least 20 seconds especially after you have been in a public place, or after blowing your nose, coughing, or sneezing.    

 Put distance between yourself and other people if Covid-19 is spreading in your community.    If you are sick: You should wear a face mask when you are around other people and before you enter a healthcare provider's office.

control_attention_ch According to the information above, which of the following IS NOT true?

- Covid-19 has not previously been seen in humans (1)
- The best way to prevent illness is to avoid being exposed to Covid-19 (2)
- You should wear a face mask around others if you are sick (3)
- If you use hand sanitizer, it should be 95% alcohol (4)

| Page Break |  |
| --- | --- |

control_boxes
Can you think of other contagious diseases that, like Covid-19, are contagious and spread from person to person? What are their symptoms?

- Contagious disease #1 (1) ________________________________________________
- Symptom (2) ________________________________________________
- Contagious disease #2 (3) ________________________________________________
- Symptom (4) ________________________________________________
- Contagious disease #3 (5) ________________________________________________
- Symptom (6) ________________________________________________
- Contagious disease #4 (7) ________________________________________________
- Symptom (8) ________________________________________________
- Contagious disease #5 (9) ________________________________________________
- Symptom (10) ________________________________________________

End of Block: Control Condition I
