# Supplementary material for: Think about your friends and family: The disparate impacts of relationship-centered messages on privacy concerns, protective health behavior, and vaccination against Covid-19
Source: PLoS One. 2022 Jul 21;17(7):e0270279. doi: 10.1371/journal.pone.0270279 (PMC9302763; doi:10.1371/journal.pone.0270279)
Supplement: S3 Fig — (DOCX) [file pone.0270279.s008.docx]

Fig A3: Conditions for Hardship Message Experiment

**Control Condition**

Start of Block: Control Condition

| 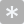 |
| --- |

control_condition_1 You have likely heard about COVID-19, the disease caused by coronavirus SARS-CoV-2 (hereafter “covid-19”).    
Can you think of other contagious diseases that, like covid-19, are contagious and spread from person to person? What are their symptoms?

**Name up to 5 other contagious diseases that spread from person to person, and one symptom of each.**

- Contagious disease 1 (4) ________________________________________________
- Symptom (5) ________________________________________________

| 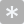 |
| --- |

control_condition_2 Can you think of another contagious disease?

- Contagious disease 2 (4) ________________________________________________
- Symptom (9) ________________________________________________

control_condition_3 Can you think of another contagious disease?

- Contagious disease 3 (4) ________________________________________________
- Symptom (9) ________________________________________________

control_condition_4 Can you think of another contagious disease?

- Contagious disease 4 (4) ________________________________________________
- Symptom (9) ________________________________________________

control_condition_5 Can you think of another contagious disease?

- Contagious disease 5 (4) ________________________________________________
- Symptom (9) ________________________________________________

| Page Break |  |
| --- | --- |

Q4 **Please review the following information about covid-19, excerpted from the CDC.**
 COVID-19 is a new disease, caused by a novel (or new) coronavirus that has not previously been seen in humans. 

 Current symptoms reported for patients with COVID-19 have included mild to severe respiratory illness with fever, cough, and difficulty breathing. 

 The best way to prevent illness is to avoid being exposed to this virus. The virus is thought to spread mainly from person-to-person.

 Protect yourself, protect others

 Stay home if you are sick, except to get medical care.

 Cover your mouth and nose with a tissue when you cough or sneeze or use the inside of your elbow.

 Wash your hands often with soap and water for at least 20 seconds especially after you have been in a public place, or after blowing your nose, coughing, or sneezing.

 Put distance between yourself and other people if COVID-19 is spreading in your community.

 If you are sick: You should wear a face mask when you are around other people and before you enter a healthcare provider's office.

| 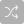 |
| --- |

According to the information above, which of the following IS NOT true?

- COVID-19 has not previously been seen in humans (1)
- The best way to prevent illness is to avoid being exposed to COVID-19 (2)
- You should wear a face mask around others if you are sick (3)
- If you use hand sanitizer, it should be 95% alcohol (4)

End of Block: Control Condition

Prime Condition

Start of Block: Prime Condition

| 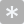 |
| --- |

prime_condition_1 You have likely heard about COVID-19, the disease caused by coronavirus SARS-CoV-2 (hereafter “ covid-19”).    
Many people have lost their jobs, temporarily or permanently, over the past month due to efforts to combat covid-19. **Can you think of anyone experiencing financial hardship due to covid**-19**, who you’d like to help?**
**In the boxes below, please list your relationship to people who are experiencing financial hardship (ex. "mother," "cousin," "friend"), and how they've been affected (ex. "laid off," "reduced wages," "fewer job opportunities").**

- Person you would help 1 (4) ________________________________________________
- How have they been affected? (5) ________________________________________________

| 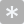 |
| --- |

prime_condition_2 Who else would you help?

- Person you would help 2 (1) ________________________________________________
- How have they been affected? (2) ________________________________________________

prime_condition_3 Who else would you help?

- Person you would help 3 (1) ________________________________________________
- How have they been affected? (2) ________________________________________________

prime_condition_4 Who else would you help?

- Person you would help 4 (1) ________________________________________________
- How have they been affected? (2) ________________________________________________

prime_condition_5 Who else would you help?

- Person you would help 5 (1) ________________________________________________
- How have they been affected? (2) ________________________________________________

| Page Break |  |
| --- | --- |

Q8 **Please review the following information about the economic impact of covid**-19**efforts** (excerpted from The New York Times, originally published April 23, 2020)**.**

Nearly a month after Washington rushed through an emergency package to aid jobless Americans, millions of laid-off workers have still not been able to apply for those benefits — let alone receive them — because of overwhelmed state unemployment systems.

...On Thursday, the Labor Department reported that another 4.4 million people filed initial unemployment claims last week, bringing the five-week total to more than 26 million.

“At all levels, it’s eye-watering numbers,” Torsten Slok, chief international economist at Deutsche Bank Securities, said. Nearly one in six American workers has lost a job in recent weeks.

Delays in delivering benefits, though, are as troubling as the sheer magnitude of the figures, he said. Such problems not only create immediate hardships, but also affect the shape of the recovery when the pandemic eases.

| 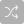 |
| --- |

According to the information above, which of the following IS NOT true?

- 26 million people have filed unemployment claims (1)
- Nearly 1 in 6 American workers has lost a job in recent weeks (2)
- Some laid-off workers have not received unemployment benefits (3)
- The article claims it it possible to know when people will return to work (4)
